# Reducing Radiation Exposure to Patients and Staff During Routine Ureteroscopic Stone Surgery: Adopting a Fluoroless Technique

**DOI:** 10.7759/cureus.16279

**Published:** 2021-07-09

**Authors:** Kellan clark, Scott King, Apexa Patel, Sharon Hill, Samuel Deem, Nathan E Hale

**Affiliations:** 1 Urology, Charleston Area Medical Center, Charleston, USA; 2 Urology, Charleston Area Medical Center (CAMC) Health Education and Research Institute Inc., Charleston, USA

**Keywords:** fluoroless, ureteroscopy, urolithiasis

## Abstract

Purpose

Urologists have an obligation to limit radiation exposure during routine stone surgery. We therefore sought to evaluate the impact of our technique for fluoroless ureteroscopy on perioperative outcomes.

Methods

Medical records of 44 patients who underwent ureteroscopy with laser lithotripsy without the use of fluoroscopy between October 2017 and December 2018 were examined. Multiple variables were collected, including age, body mass index (BMI), mean stone volume and density, operative times, complications, and stone-free rates. These patients were then compared to a cohort of 44 patients who underwent stone surgery with a conventional technique prior to the adoption of a fluoroless technique by the same surgeons. The primary study outcome was reduction of intraoperative fluoroscopy. Secondary outcomes included complications, operative time, and stone-free rates.

Results

Of the 44 patients undergoing a fluoroless technique, 38 (86.4%) were able to receive ureteroscopy without the use of fluoroscopy. A significant difference was observed in mean fluoroscopy times for the fluoroless group (2.8 seconds) and the conventional group (33.7 seconds). No complications were observed in either group. Operative length was 38.9 minutes in the fluoroless group versus 42.2 minutes in the conventional group. Age, BMI, stone characteristics, and stone-free rates were similar in both.

Conclusions

The use of a fluoroless technique for the treatment of uncomplicated stones is not only safe but also effective and efficient. This technique eliminates extraneous radiation doses to the patient and operative staff in most cases.

## Introduction

Urolithiasis is currently estimated to affect 10.6% of men and 7.1% of women in the United States, with an increased lifetime risk of a symptomatic stone episode [1]. Urolithiasis often results in the need for an intervention. These patients are exposed to potentially harmful doses of ionizing radiation starting at diagnosis and continuing throughout their treatment and follow-up. Many imaging modalities, which include computed tomography (CT) and fluoroscopy, are used for the detection of stones and help guide treatment [2]. In addition, fluoroscopy helps identify complications intraoperatively. Unfortunately, fluoroscopy use during these procedures exposes the patient, operative staff, and surgeon to ionizing radiation. All forms of ionizing radiation have the potential to cause cancer, with no standard limitation for prevention [3-5]. Although this topic remains heavily debated within the medical physics community, the principles of “justification” and “optimization” applied to the use of ionizing radiation for medical purposes remain foundational among clinicians.

Various measures are routinely employed to reduce radiation exposure during fluoroscopy. Examples include utilizing lead shielding and employing single-pulse per second images [6]. In addition, alternative subspecialties are developing techniques to reduce the use of intraoperative fluoroscopy, including cardiology, vascular, and neurosurgery, orthopedics, radiology, and others [7-9]. In a similar fashion, urologists are pursuing methods in order to lower the amount of radiation used during ureteroscopy. Described interventions include surgeon education, C-arm aiming lasers, fluoroscopy settings, use of ultrasound, smaller instruments, and new procedural techniques [6,10-13]. There have been several techniques described in the literature aimed at reducing the usage of fluoroscopy during routine ureteroscopic stone surgery [2,10-15]. Most recently, a group of urologists described ureteroscopy without fluoroscopy and reported a similar complication rate and operative time in patients undergoing conventional ureteroscopy [2].

Inspired by prior efforts to reduce radiation exposure, we elected to proceed with the implementation of a fluoroless technique for the treatment of urolithiasis. The purpose of this study was to further substantiate the feasibility, safety, and efficacy of a fluoroless ureteroscopic stone surgery (FUSS) technique in the management of uncomplicated ureteral or renal stones. In addition, we sought to develop criteria for patient selection and to compare outcomes to those patients managed with intraoperative use of fluoroscopic imaging.

## Materials and methods

**Figure 1:**
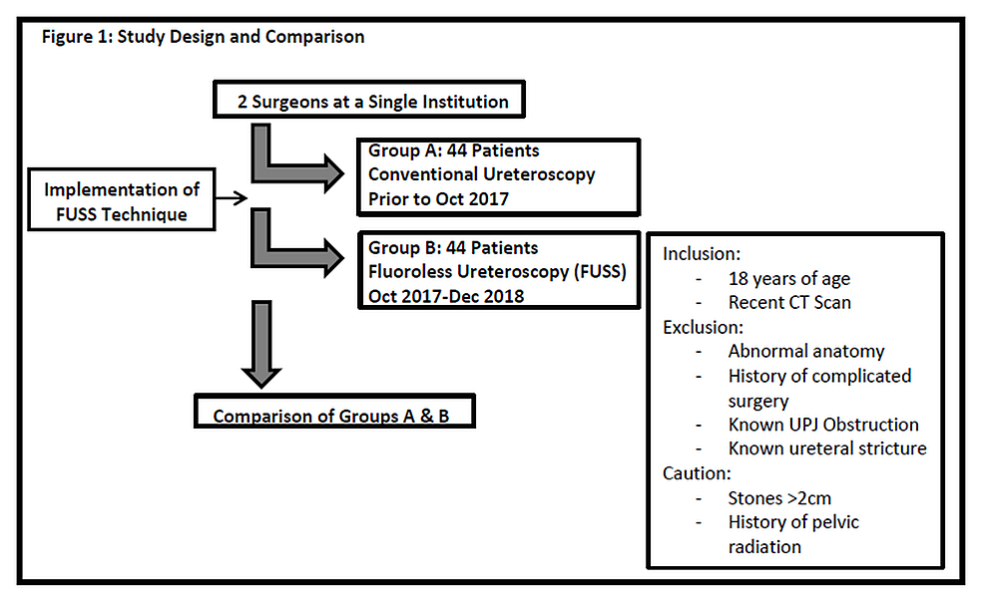
Study design and comparison FUSS, fluoroless ureteroscopic stone surgery; UPJ, ureteropelvic junction

Institutional Review Board approval was obtained to retrospectively review who underwent stone surgery between October 2017 and December 2018. During the study period, 44 patients underwent FUSS performed by two surgeons at a single academic institution. The technique was applied to patients deemed to be low risk for complications based on inclusion and exclusion criteria. Inclusion criteria included patients at least 18 years of age with a CT scan performed within six weeks from the time of surgery and available for review. Exclusion criteria were urinary tract abnormalities on imaging (i.e. duplicated collecting system) and a history of prior complicated stone or ureteral surgery for stricture or ureteropelvic junction obstruction (UPJO). Additional caution was exercised for the patients with a stone size of greater than 2 mm and patients with a history of pelvic radiation. If these criteria were not met, the surgeons opted to proceed with a conventional technique utilizing fluoroscopy. Emphasis was placed on aborting the fluoroless approach if encountering any difficulty. The comparison group was created by matching the same inclusion and exclusion criteria to identify patients who underwent conventional ureteroscopic stone treatment by the same surgeons prior to the implementation of FUSS (Figure 1). All ureteroscopy was performed using a Karl Storz Flexible Uretero-Renoscope Flex-X^C^ (Karl Storz, Tuttlingen, Germany).

The FUSS technique was executed beginning with routine cannulation of the ureteral orifice and insertion of guide and safety wires. Tactile cues were noted, suggesting routine progression into the renal pelvis. An ureteroscope was advanced along a guidewire under direct visualization. The laser lithotripsy was then performed to treat the stone. The ureteroscope was then withdrawn, marking the ureteroscope at the ureteropelvic and ureterovesical junctions, as well as evaluating the ureter to ensure that no ureteral stone or debris was left untreated. The stent was then placed under cystoscopic direct visualization, and a single flash of fluoroscopy was used to confirm a satisfactory position based on the appropriate curl of the ureteral stent.

The primary outcome of the study was the total time reduction of intraoperative fluoroscopy. Secondary outcomes included a stone-free rate based on postoperative CT or renal ultrasound (using ≤3 mm criteria) within eight weeks, operative time, complications, and re-treatment rates. In addition, the implementation of the fluoroless technique within a residency training program was under particular subjective evaluation.

Demographic, operative, and clinical characteristics were compared between the patients who received FUSS or conventional ureteroscopy. A descriptive analysis of the study groups was performed using means, standard deviation, ranges, and percentages. Continuous variables were analyzed using Student’s t-test, and categorical variables were analyzed using Fisher’s exact test, with p < 0.05 considered statistically significant. Statistical analyses were performed using SAS 9.3 (SAS Institute, Cary, NC).

## Results

The demographic and operative characteristics of the study groups are summarized in Table 1. A total of 44 patients underwent ureteroscopy utilizing the FUSS technique. The average patient was 52.5 years old, with slight male predilection and a body mass index (BMI) of 31.2. Fourteen patients had indwelling stents, and the mean stone volume was 578.5 mm^3^ with a mean density of 819 Hounsfield units (HU). No statistical difference was identified in patient age, sex, BMI, stone density or volume, or prior stent placement between the fluoroless and conventional groups.

**Table 1:** Demographics and clinical characteristics BMI, body mass index; FUSS, fluoroless ureteroscopic stone surgery

	FUSS group (n=44)	Conventional group (n=44)	p-Value
Age (years), mean ± SD	52.5 ± 18.0	53.8 ± 15.9	0.721
Gender, n (%)			
Male	23 (52.3%)	26 (59.1%)	0.519
Female	21 (47.7%)	18 (40.9%)	
BMI, mean ± SD	31.2 ± 6.2	30.4 ± 6.8	0.587
Mean stone volume (mm^3^), mean ± SD	578.5 + 678.1	553.7 ± 325.1	0.859
Mean stone density (HU), mean ± SD	819.0 ± 296.0	919.3 ± 350.9	0.150
Prior stent, n (%)	14 (31.8%)	11 (25.0%)	0.478
Hydronephrosis, n (%)	39 (88.6%)	29 (65.9%)	
Mild	20 (51.3%)	11 (37.9%)	0.011
Moderate	17 (43.6%)	16 (55.2%)	
Severe	2 (5.1%)	2 (6.9%)	

**Figure 2:**
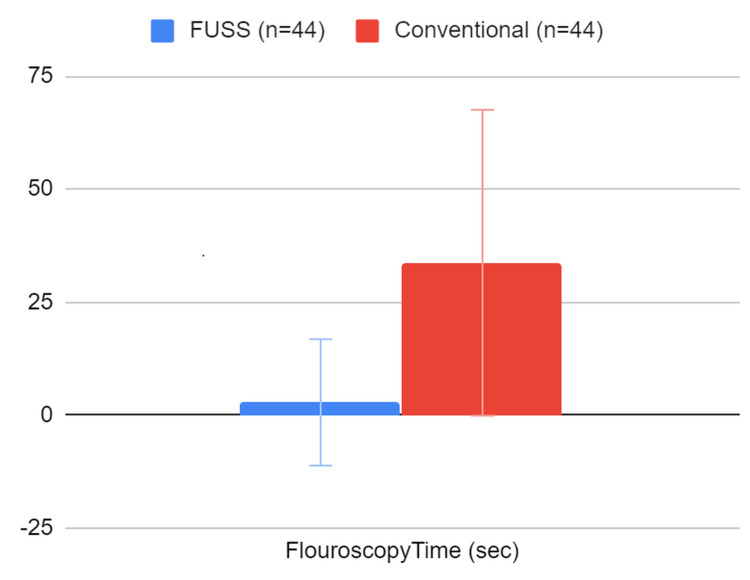
Fluoroscopy times (Student’s t-test) FUSS, fluoroless ureteroscopic stone surgery

There was a significant difference in mean fluoroscopy times for the fluoroless group (2.8 seconds) and the conventional group (33.7 seconds) using Student’s t-test (Figure 2). The 2.8 seconds was used as a confirmatory measure to ensure satisfactory stent placement as a part of the learning process. This was continued for consistency, although unnecessary by the end of the study, as the involved surgeons felt comfortable with fluoroless stent placement. Of the 44 fluoroless cases, six (13.6%) cases were unable to be completed, and the technique was aborted due to difficult access in three cases. Despite conversion to conventional ureteroscopy, these patients and respective data remained within the FUSS group based on intention to treat. There was no difference in operative times, intraoperative complications, the requirement for the subsequent procedure, or postoperative stone-free rate. Primary and secondary outcomes are summarized in Table 2.

**Table 2:** Primary and secondary outcomes FUSS, fluoroless ureteroscopic stone surgery

	FUSS group (n=44)	Conventional group (n=44)	p-Value
Fluoroscopy time (seconds), mean ± SD	2.8±10.5	33.7 ± 33.9	<0.0001
Operative time (minutes), mean ± SD	38.9±20.5	42.2±16.3	0.407
Intraoperative complications	0	0	1
Stone-free rate, n (%)	27 (84.4%)	31 (86.1)	0.840
Subsequent procedure	0	0	1

## Discussion

Our study showed an effective way to safely treat kidney stones with ureteroscopy while minimizing radiation exposure. With an increasing incidence of nephrolithiasis, it becomes increasingly important to optimize complication-free and stone-free rates while minimizing radiation exposure to patients and medical personnel. This is further substantiated when considering the increasing incidence of nephrolithiasis and recurrence as well as rates of surgical intervention [16-17].

A recent study developed a model to predict the incidence of secondary malignancy secondary to radiation exposure in the management of nephrolithiasis. The lifetime attributable risk of malignancy was as low as 0.064% in males over 70 years of age and as high as 0.39% in women aged 20-30 years based on a higher incidence of uterine and ovarian pathology. While the risk for a single individual is small, the model suggests a national incidence of 862.7 cases of radiation-induced secondary malignancy, leading to 545.3 deaths annually [18].

Varying opinions do exist regarding the carcinogenic threshold of medical radiation. Survivors of atomic bombings and nuclear power plant employees have demonstrated an increased incidence of secondary malignancy [19]. As for application to modern-day medical imaging, studies from Australia and England show an increased incidence of secondary malignancy in patients exposed to CT while in childhood or adolescence [20-21]. These studies would fall in support of the linear no-threshold hypothesis (LNTH), suggesting a direct relationship between the dose of radiation and the risk of developing secondary cancer. In contrast to the previously cited studies, critics do exist within the medical physics community, with some suggesting a health benefit of low-dose radiation. One article supporting this idea describes a cohort of British radiologists whose cancer and all-cause mortality rates were significantly lower when compared to physicians of other specialties [22]. The described cohort was from an early era benefiting from the routine use of protective shielding.

The majority of medical personnel generally ascribe to the “as low as reasonably achievable” (ALARA) principle when it comes to administering ionizing radiation. Many professional organizations and committees support the principles of “justification” and “optimization” to reduce radiation exposure. Alliances of health care coalitions such as Image Gently® and Image Wisely® are entities advocating for the safe and optimal use of radiation in children and adults. Organizations such as the International Commission on Radiological Protection (IRCP), the American Academy of Physicists in Medicine (AAPM), the International Organization for Medical Physics (IOMP), the Health Physics Society (HPS), and the Basis for Estimating risks of Ionizing Radiation (BEIR VII) report accept evidence that low doses of radiation may pose a risk, even if it is a small risk, and efforts to minimize dosage are founded [23].

Although minimizing risks to patients remains a priority, a fluoroless approach to ureteroscopy also benefits the operating room staff, anesthesia personnel, and surgeon, many of who will be exposed to several cases throughout the course of the day, week after week. At our institution, the cystoscopy suite’s X-ray tube generates an effective dose of 1.92 mSv per 60 seconds of use. Though staff members wear protective shielding, one does recognize the difference in radiation exposure between the fluoroless and conventional techniques, with mean fluoroscopy times of 2.8 versus 33.7 seconds, respectively.

Fluoroscopy reducing techniques in ureteroscopy have developed in a progressive manner. Over time, urologists have successfully performed fluoroless distal ureteroscopy to fluoroless ureteral stent placement and now fluoroless proximal ureteroscopy and retrograde intrarenal surgery [2,14-15,24]. The presented technique involved using tactile feedback, visual cues (such as externalized guidewire length and ureteroscope measurements), and direct visualization to execute the necessary steps of the procedure. A clinically significant reduction in fluoroscopy time was appreciated with unchanged operative times and rates of re-treatment, stone-free status, and complications. These findings further substantiate the feasibility and safety of the technique. Interestingly, in the six cases that were not able to be completed in a fluoroless manner, there was a mean fluoroscopy time of 20.8 seconds, a significant reduction compared to conventional usage. This further speaks to the efficacy of education and surgeon awareness of the methods of reducing intraoperative radiation.

Patient selection remains essential to the success of a fluoroless approach. Caution should be taken in adult patients with abnormalities of the urinary tract, prior complicated stone surgery or pelvic radiation, larger stones, ureteral stricture, or UPJO. Another potentially complicating factor is the presence of an impacted stone. Preoperative suspicion of an impacted stone should preclude the surgeon from proceeding in a fluoroless manner.

Another important element of the study is the successful implementation within an academic setting. Previous studies have demonstrated variation of intraoperative fluoroscopy time between trainees [25]. Additionally, protocols for radiation education have been shown to reduce intraoperative fluoroscopy time when compared to the same surgeon’s previous cases as well as when compared to experienced surgeons who did not receive similar training [10]. Due to the importance of understanding tactile cues, senior urology residents and attending surgeons were the primary operators in this study. Despite involvement by upper level trainees, there was no increase in peri- or postoperative complications, and the stone-free rate was unchanged. Setting a foundation of radiation stewardship in the academic environment is an important step towards reducing patient, staff, and surgeon exposure for generations to come.

Limitations include the retrospective nature of the study along with the potential for selection bias as to who was performing the case when/if the decision to abort the fluoroless approach was made. An additional limitation is that postoperative imaging was not standardized among the fluoroless and conventional cohorts. However, practice patterns were unchanged among those physicians involved in the study in this regard. Measurement bias is another potential bias as the fluoroscopy exposure was not blinded.

One randomized controlled trial has been conducted to date for fluoroless distal ureteroscopy [14]. Future randomized controlled trials comparing proximal ureteroscopy and intrarenal cases to controls with standardized postoperative imaging would further progress the understanding and acceptance of fluoroless urological endoscopic surgery.

## Conclusions

Adopting a fluoroless technique for the endoscopic management of uncomplicated ureteral and renal urolithiasis appears feasible and safe, and led to similar patient outcomes compared to conventional ureteroscopy while eliminating the risks of radiation exposure. In addition, it was well received by residents in training who demonstrated aptitude by executing the technique in 38/44 patients. Fluoroless ureteroscopy remains reserved for uncomplicated stone cases, and patient selection is imperative. Variance from the routine technique should prompt the surgeon in the use of fluoroscopy to assure patient safety, minimize the risk of complications, and achieve similar postoperative outcomes.

## References

[1] Scales CD Jr, Smith AC, Hanley JM, Saigal CS (2012). Prevalence of kidney stones in the United States. Eur Urol.

[2] Olgin G, Smith D, Alsyouf M (2015). Ureteroscopy without fluoroscopy: a feasibility study and comparison with conventional ureteroscopy. J Endourol.

[3] Wambani JS, Korir GK, Tries MA, Korir IK, Sakwa JM (2014). Patient radiation exposure during general fluoroscopy examinations. J Appl Clin Med Phys.

[4] Preston DL, Pierce DA, Shimizu Y, Cullings HM, Fujita S, Funamoto S, Kodama K (2004). Effect of recent changes in atomic bomb survivor dosimetry on cancer mortality risk estimates. Radiat Res.

[5] Cardis E, Vrijheid M, Blettner M (2007). The 15-Country Collaborative Study of Cancer Risk among Radiation Workers in the Nuclear Industry: estimates of radiation-related cancer risks. Radiat Res.

[6] Yecies TS, Fombona A, Semins MJ (2017). Single pulse-per-second setting reduces fluoroscopy time during ureteroscopy. Urology.

[7] Kuon E, Glaser C, Dahm JB (2003). Effective techniques for reduction of radiation dosage to patients undergoing invasive cardiac procedures. Br J Radiol.

[8] Gebhard FT, Kraus MD, Schneider E, Liener UC, Kinzl L, Arand M (2006). Does computer-assisted spine surgery reduce intraoperative radiation doses?. Spine (Phila Pa 1976).

[9] Kirkwood ML, Arbique GM, Guild JB, Timaran C, Chung J, Anderson JA, Valentine RJ (2013). Surgeon education decreases radiation dose in complex endovascular procedures and improves patient safety. J Vasc Surg.

[10] Weld LR, Nwoye UO, Knight RB (2014). Safety, minimization, and awareness radiation training reduces fluoroscopy time during unilateral ureteroscopy. Urology.

[11] Danilovic A, Nunes E, Lipkin ME (2019). Low dose fluoroscopy during ureteroscopy does not compromise surgical outcomes. J Endourol.

[12] Greene DJ, Tenggadjaja CF, Bowman RJ, Agarwal G, Ebrahimi KY, Baldwin DD (2011). Comparison of a reduced radiation fluoroscopy protocol to conventional fluoroscopy during uncomplicated ureteroscopy. Urology.

[13] Singh V, Purkait B, Sinha RJ (2016). Prospective randomized comparison between fluoroscopy-guided ureteroscopy versus ureteroscopy with real-time ultrasonography for the management of ureteral stones. Urol Ann.

[14] Mohey A, Alhefnawy M, Mahmoud M, Gomaa R, Soliman T, Ahmed S, Noureldin YA (2018). Fluoroless-ureteroscopy for definitive management of distal ureteral calculi: randomized controlled trial. Can J Urol.

[15] Hsi RS, Harper JD (2013). Fluoroless ureteroscopy: zero-dose fluoroscopy during ureteroscopic treatment of urinary-tract calculi. J Endourol.

[16] Fahmy NM, Elkoushy MA, Andonian S (2012). Effective radiation exposure in evaluation and follow-up of patients with urolithiasis. Urology.

[17] Ferrandino MN, Bagrodia A, Pierre SA, Scales CD Jr, Rampersaud E, Pearle MS, Preminger GM (2009). Radiation exposure in the acute and short-term management of urolithiasis at 2 academic centers. J Urol.

[18] Yecies TS, Semins MJ (2019). Modeling the incidence of secondary malignancy related to ionizing radiation use in the management of nephrolithiasis. Urology.

[19] Pierce DA, Preston DL (2000). Radiation-related cancer risks at low doses among atomic bomb survivors. Radiat Res.

[20] Mathews JD, Forsythe AV, Brady Z (2013). Cancer risk in 680,000 people exposed to computed tomography scans in childhood or adolescence: data linkage study of 11 million Australians. BMJ.

[21] Pearce MS, Salotti JA, Little MP (2012). Radiation exposure from CT scans in childhood and subsequent risk of leukaemia and brain tumours: a retrospective cohort study. Lancet.

[22] Cameron JR (2002). Radiation increased the longevity of British radiologists. Br J Radiol.

[23] National Research Council (2006). Health Risks from Exposure to Low Levels of Ionizing Radiation: BEIR VII Phase 2. Health Risks from Exposure to Low Levels of Ionizing Radiation: BEIR VII Phase 2.

[24] Brisbane W, Smith D, Schlaifer A, Anderson K, Baldwin DD (2012). Fluoro-less ureteral stent placement following uncomplicated ureteroscopic stone removal: a feasibility study. Urology.

[25] Elkoushy MA, Andonian S (2013). Variations among urology trainees in their use of fluoroscopy during ureteroscopy. J Endourol.

